# The StW 573 Little Foot Fossil Should Not Be Attributed to *Australopithecus prometheus*

**DOI:** 10.1002/ajpa.70177

**Published:** 2025-11-29

**Authors:** Jesse M. Martin, Luca Morris‐Obst, A. B. Leece, Stephanie Baker, Andy I. R. Herries, David S. Strait

**Affiliations:** ^1^ Leverhulme Centre for Human Evolutionary Studies, Department of Archaeology University of Cambridge Cambridge UK; ^2^ Palaeoscience Labs, Department of Archaeology and History Latrobe University Bundoora Victoria Australia; ^3^ Palaeo‐Research Institute University of Johannesburg Johannesburg South Africa; ^4^ Department of Anthropology Washington University in St Lous St Louis Missouri USA

**Keywords:** *Australopithecus africa*nus, *Australopithecus prometheus*, human evolution, Sterkfontein

## Abstract

**Objectives:**

To test the hypothesis that the StW 573 (Little Foot) fossil specimen should be attributed taxonomically to *Australopithecus prometheus*.

**Materials and Methods:**

We adopt the methods of classic morphology by comparing StW 573 to the type specimen of *A. prometheus* (MLD 1) and other consensus members of *Australopithecus africanus*. We utilize qualitative anatomical descriptions and comparisons, supplemented with the examination of selected relevant quantitative measurements.

**Results:**

We find that the morphology preserved by StW 573 does not support assigning that specimen to *A. prometheus* because it does not share a unique suite of primitive and derived traits in common with the *A. prometheus* type specimen, MLD 1. Specifically, StW 573 differs from MLD 1 in having a more pronounced external occipital protuberance, a sagittal crest at lambda, an asterionic notch, a long nuchal plane, and a smaller cranial capacity. Regarding these same areas of anatomy, MLD 1 more closely resembles Sts 5, and MLD 37/38, consensus members of *A. africanus* .

**Discussion:**

*A. prometheus* should remain a junior synonym for *A. africanus* based on the demonstrated morphological similarities between MLD 1 and the broader *A. africanus* sample. Conversely, while StW 573 cannot be attributed to *A. prometheus*, the results of this study indicate that it also differs in meaningful ways from specimens conventionally attributed to *A. africanus* .

## 1 Introduction

The StW 573 (Little Foot) fossil from Sterkfontein in South Africa is one of the most complete and best‐preserved australopiths in the hominin record. StW 573 is the first and only hominin discovered from within the Silberberg Grotto (Clarke and Tobias 1995; Clarke 2008; Clarke and Kuman 2019) in Member 2 (as originally defined by Partridge (1978) and Partridge and Watt (1991)). Prior to the fossil having been completely removed from breccia, Clarke (1998, 2008) indicated that he considered StW 573 to differ morphologically from *Australopithecus africanus* and to probably belong to a different species. In their seminal description of StW 573's skull, Clarke and Kuman (2019) align StW 573 with a group of specimens from Sterkfontein Member 4 and Makapansgat Limeworks Member 3 (including the type specimen of *Australopithecus prometheus*, MLD 1) and assign these to *A. prometheus*. Central to Clarke and Kuman's (2019) interpretation of StW 573 and the broader Makapansgat Limeworks + Sterkfontein assemblage are two hypotheses: (1) There are two distinct gracile australopith morphotypes (species) represented within the combined Sterkfontein + Makapansgat Limeworks assemblage and (2) one of these morphotypes (that including StW 573) shares its closest affinities with MLD 1, the type specimen of *A. prometheus*. It is a subtle but non‐trivial point to notice that these two hypotheses are independent and can therefore be tested separately. This analysis is concerned with the second of Clarke and Kuman's (2019) hypotheses, namely, whether StW 573 shares affinities with the type specimen of *A. prometheus*.

Dart (1948) erected *A. prometheus* to accommodate the fossils recovered from Makapansgat Limeworks because he considered them to differ from *Australopithecus africanus* (at the time represented only by the Taung child) and *Plesianthropus transvaalensis* from Sterkfontein Member 4 (now generally subsumed within *A. africanus* ) in ways likely to represent species‐level differences. Dart (1948) differentiated *A. prometheus* from *A. africanus* (and all other known hominin species) based on the presence of multiple Wormian bones along the lambdoid suture in MLD 1, and a cranial capacity that was larger than that observed in the hominins from Sterkfontein Member 4 and Taung. However, Robinson (1954) systematically compared all South African hominins and assigned specimens from Taung, Sterkfontein, and Makapansgat Limeworks to a single species, *Australopithecus africanus*, and for nearly 70 years this taxonomy has been widely accepted, though not unchallenged. While a small number of researchers (Lockwood and Tobias 2002; Moggi‐Cecchi et al. 2006; Grine 2013) have noted the large degree of variability and potential for two species at Sterkfontein, only Clarke (1985, 1988, 2008, 2013), Clarke and Tobias (1995), and Clarke and Kuman (2019) have consistently split the Makapansgat Limeworks + Sterkfontein australopith sample into two distinct species, *A. africanus* and *A. prometheus* (e.g., Clarke 1988, 2013; Clarke and Kuman 2019).

Logically, to be considered the same species, MLD 1 and StW 573 should share a unique suite of primitive and derived characters that distinguish them from all other known hominin taxa. Problematically, the type specimen of *A. prometheus* (MLD 1) is an occipito‐parietal fragment that preserves only limited diagnostic morphology, and so comparison between the almost complete StW 573 skull is necessarily delimited by the morphology that overlaps between the two specimens. In their taxonomic summary, Clarke and Kuman (2019) provide a list of characters that they argue define *A. prometheus* and differentiate it from *A. africanus*; however only three of these traits are preserved and/or assessable on the *A. prometheus* type specimen MLD 1. These traits are a cranial capacity above 500 cc in males, a sagittal crest in males, and a parietal bone whose lateral wall is straight and vertically oriented in posterior view rather than inclined. It is relevant to note that Clarke and Kuman (2019) do not address one of the two traits Dart (1948) argued defines *A. prometheus* which is the presence of a complex lambdoidal suture characterized by Wormian bones. While we agree with Robinson (1954) that this trait is unlikely to have taxonomic valence, it is nonetheless one of only two morphological characteristics Dart (1948) offered towards the morphological diagnosis of *A. prometheus* and is absent in StW 573. In any case, if StW 573 does not share a unique suite of primitive and derived character states with MLD 1 then the allocation of StW 573 to *Australopithecus prometheus* is unsupported, regardless of whether there are two morphotypes within the combined Sterkfontein + Makapansgat assemblage. We test this hypothesis by comparing the occipito‐parietal morphology of StW 573 and MLD 1.

## 2 Materials and Methods

We adopt the methods of classical morphology, which are appropriate given that such methods have previously been used both to define *A. prometheus* and to assess the taxonomic affinities of StW 573 (Clarke and Kuman 2019). As a generalization, these entail qualitative descriptions and comparisons paired with examination of selected measurements (linear dimensions and ratios of such dimensions) that quantify aspects of morphology that empirically vary qualitatively. Moreover, in this particular study, statistical analysis using geometric morphometrics is of limited utility because so few landmarks are preserved on MLD 1 and because the StW 573 cranium is distorted in the region of these landmarks (including distortion to surfaces and curvilinear features; see fig. 7 in Clarke and Kuman (2019)). Distortion would likewise influence landmark‐free methods. StW 573 preserves aspects of morphology (including linear dimensions) that have taxonomic valence; however a geometric morphometric analysis in the absence of a complete cranial retro‐deformation would be uninformative and unfair to the position taken by Clarke and Kuman (2019) because such distortion would inappropriately exaggerate the difference between StW 573 and other specimens. Such a retro‐deformation is beyond the scope of this study, and in any case would complement but not supersede the specific details of anatomy used by Clarke and Kuman (2019) to make their taxonomic argument.

We examine seven morphological traits preserved on MLD 1. Five of these were examined by Clarke and Kuman (2019); lateral braincase wall orientation, nuchal plane orientation, nuchal plane length, configuration of the temporal lines/sagittal crest, and cranial capacity (note that the two nuchal plane traits were not included in their list of traits diagnosing *A. prometheus*). We additionally examine two traits not considered by them: expression of the external occipital protuberance, and the configuration of the asterionic region.

We compare MLD 1 and StW 573 to other South African hominin specimens preserving occipito‐parietal morphology that were discussed by Clarke and Kuman (2019), StW 53, Sts 5, StW 252. We further add measurements for specimen MLD 37/38 which Clarke and Kuman (2019) did not measure but attributed to *A. africanus* . Sts 5 is nearly universally recognized as *A. africanus* following Robinson (1954), and there is similarly broad agreement that MLD 37/38 likewise samples this species. MLD 1 and StW 252 are conventionally attributed to *A. africanus*; however Clarke (1985, 1988, 2008, 2013), Clarke and Kuman (2019) and Clarke and Kuman (2019) attribute them to *A. prometheus*. StW 53 is conventionally attributed to *Homo* (e.g., Tobias 1978), but Clarke and Kuman (2019) assign it to *A. africanus* .

**FIGURE 1:**
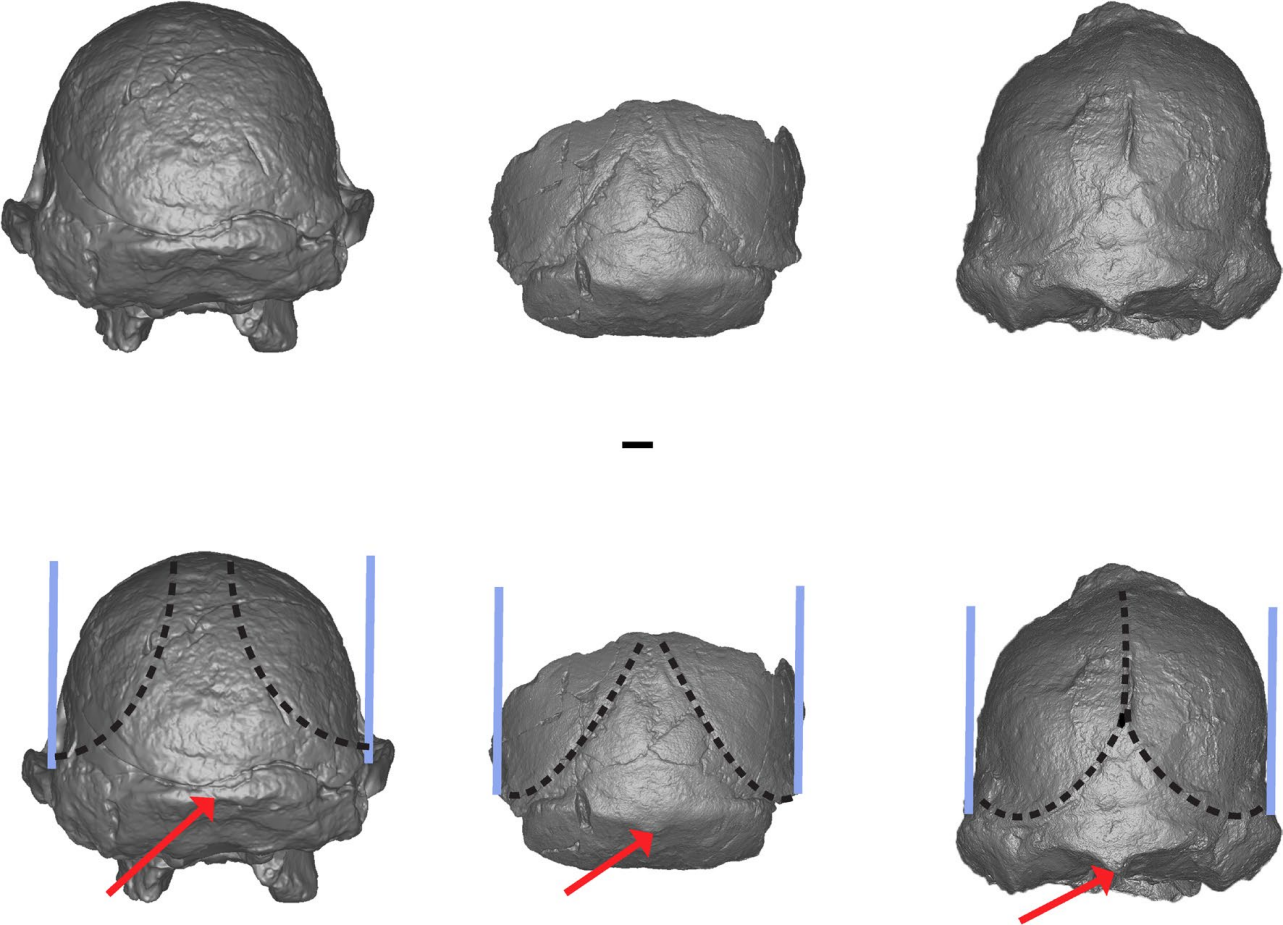
Sts 5, MLD 1, StW 573 positioned in Frankfurt Horizontal in posterior view. Black dotted line demonstrates the configuration of the superior temporal lines, the red arrow indicates the configuration of the external occipital protuberance, and the blue line demonstrates the slope of the parietals relative to a vertical plane.

The original fossil specimens StW 573, Sts 5, and MLD 1 and the Clarke reconstruction of StW 53 were scanned using an Artec Space Spider Scanner to a resolution of 300 μm. Images presented in this paper are derived from these scans. To orient MLD 1 in approximately Frankfurt Horizontal for the purposes of generating Figure 1, the specimen was aligned with Sts 5 using overlapping morphology and then rotated into Frankfurt Horizontal using the landmarks preserved by Sts 5.

Metrical data presented in this paper were taken after Clarke and Kuman (2019) on original fossil specimens (StW 573, Sts 5, MLD 1, MLD 37/38) and on casts of Clarke's reconstruction (StW 53) using digital calipers. All measurements were taken three times and averaged to control for intra‐observer error.

## 3 Results

Key morphological observations by Clarke and Kuman (2019) were provided in their fig. 12, which depicts pronounced differences in lateral braincase wall orientation between their proposed *A. prometheus* (MLD 1, StW 252, StW 573) and *A. africanus* (Sts 5, StW 53) morphotypes when seen in posterior view, with the former exhibiting a more vertical wall than the latter. In our view, this figure does not have some of the fossil specimens correctly aligned in Frankfurt Horizontal. We note that in their fig. 12C,D, Sts 5 and StW 53 appear to have been rotated antero‐inferiorly out of Frankfurt Horizontal to such an extent that the outline of the foramen magnum is clearly visible, and the nuchal line is at mid‐cranial height. Rotating these two specimens in this way has the effect of making the walls of the parietals appear to be strongly sloped. Here we present an image of StW 573, MLD 1, and Sts 5, in Frankfurt Horizontal from posterior view. This orientation (Figure 1) removes much of the impetus for supposing that there are meaningful differences in the configuration of the parietal bones and the posterior neurocranial profile between Sts 5, MLD 1, and StW 573. Contra Clarke and Kuman (2019), when placed in Frankfurt Horizontal and viewed posteriorly, the parietal bones in StW 573, Sts 5, and MLD 1 are broadly similar with vertical/subvertical lateral braincase walls (Figure 1).

The slope of the lateral braincase walls can be quantified simply using a ratio defined as breadth across the mid‐parietals divided by braincase breadth above the supramastoid crest, where a value of 1.0 indicates a vertical wall and a value less than 1.0 indicates walls that slope superomedially. Clarke and Kuman (2019: tab. 6) collected these linear measurements, and ratios calculated using their values (Table 1) indicate, indeed, that the lateral braincase wall appears to be more vertical in the specimens they attribute to *A. prometheus* (StW 573, StW 252, MLD 1) than in specimens they attribute to *A. africanus* (Sts 5, StW 53). We attempted to duplicate these measurements with digital calipers, with the understanding that a lack of well‐defined landmarks would introduce error into any comparison between our measurements and theirs (Clarke and Kuman 2019). We found broadly similar values (within a few percentage points) for “Braincase breadth above supramastoid crest” in all specimens, and for “Braincase breadth across mid‐parietals” in specimens StW 573, StW 252, and MLD 1. However, we found that Sts 5 and Clarke's reconstruction of StW 53 were notably broader across the parietals than recorded by Clarke and Kuman (2019). We have no explanation for this discrepancy, although the fact that most of our data were similar would appear to rule out systemic differences in how we and they collected these measurements. Regardless, our measurements and the calculated values of the braincase wall slope ratio do not support the claim that these specimens are radically different with respect to the slope of the lateral braincase wall.

**TABLE 1:** Braincase breadth and lateral wall slope, as measured by Clarke and Kuman (2019) versus the present study.

	StW 573	MLD 1	StW 252	Sts 5	StW 53^a^	MLD 37/38
Clarke and Kuman (2019)						
Braincase breadth across mid‐parietals	90	95	103	**85**	**88**	
Braincase breadth above supramastoid crest	93	98	103	100	111	
Braincase wall slope ratio^b^	0.97	0.97	1.00	**0.85**	**0.79**	
This study						
Braincase breadth across mid‐parietals	94.0	(104.4)^c^	?^d^	**94.4**	**(98.7)**	97.8
Braincase breadth above supramastoid crest	95.5	99.7	?	96.0	(109.2)	104.4
Braincase wall slope ratio^a^	0.98	1.04	?	**0.98**	**0.90**	0.96

*Note:* Key differences are highlighted in bold.

^a^ Clarke reconstruction.

^b^ Calculated as (Braincase breadth across mid‐parietals) / (Braincase breadth above supramastoid crest).

^c^ Number in parentheses are estimates.

^d^ We assess StW 252 as being too fragmentary to measure.

We agree with Clarke and Kuman (2019) that the nuchal plane of StW 573 faces more inferiorly than posteriorly which is synonymous with a relatively horizontal nuchal plane. It is difficult to assess the orientation of the nuchal plane in MLD 1 owing to its fragmentary nature, but we assess qualitatively that the nuchal plane in MLD 1 is more steeply inclined than that of StW 573 and is in this regard more like that of Sts 5 (Figure 1). The nuchal plane in StW 573 is also markedly longer (relative to braincase breadth above the supramastoid crest) than those of MLD 1 and Sts 5 (Table 2).

**TABLE 2:** Nuchal plane length relative to braincase breadth.

	StW 573	MLD 1	Sts 5	MLD 37/38
Braincase breadth above supramastoid crest	95.5	99.7	96.0	104.4
Inion‐opisthion chord	44.4	35.6	30.8	33.7
Nuchal plane length ratio^a^	0.46	0.36	0.32	0.32

^a^ Calculated as (Braincase breadth above supramastoid crest)/(Inion‐opisthion chord).

The configuration of the temporal lines differs between MLD 1 and StW 573 (Figure 1). Posteriorly, the temporal lines diverge smoothly and gently from their narrowest point in MLD 1, whereas they project more strongly inferiorly before coursing laterally in StW 573. The inferior most extent of the sagittal crest is positioned slightly above lambda in StW 573, but the point of closest convergence in MLD 1 is well above lambda and does not form a crest. While this trait could be associated with sexual dimorphism, we note that Clarke and Kuman (2019) propose that MLD 1 is likely a male whereas StW 573 is likely female, in which case sexual dimorphism would not explain the more extensively convergent temporal lines in StW 573.

Similarly, MLD 1 has a relatively gracile external occipital protuberance that compares favorably to that of Sts 5, whereas this feature is robust and well marked in StW 573 (Figure 1).

As reported by Clarke and Kuman (2019), StW 573 has a smaller cranial capacity (at least 408 cc) than MLD 1 (520–530 cc), although such a difference might not necessarily have taxonomic significance.

**FIGURE 2:**
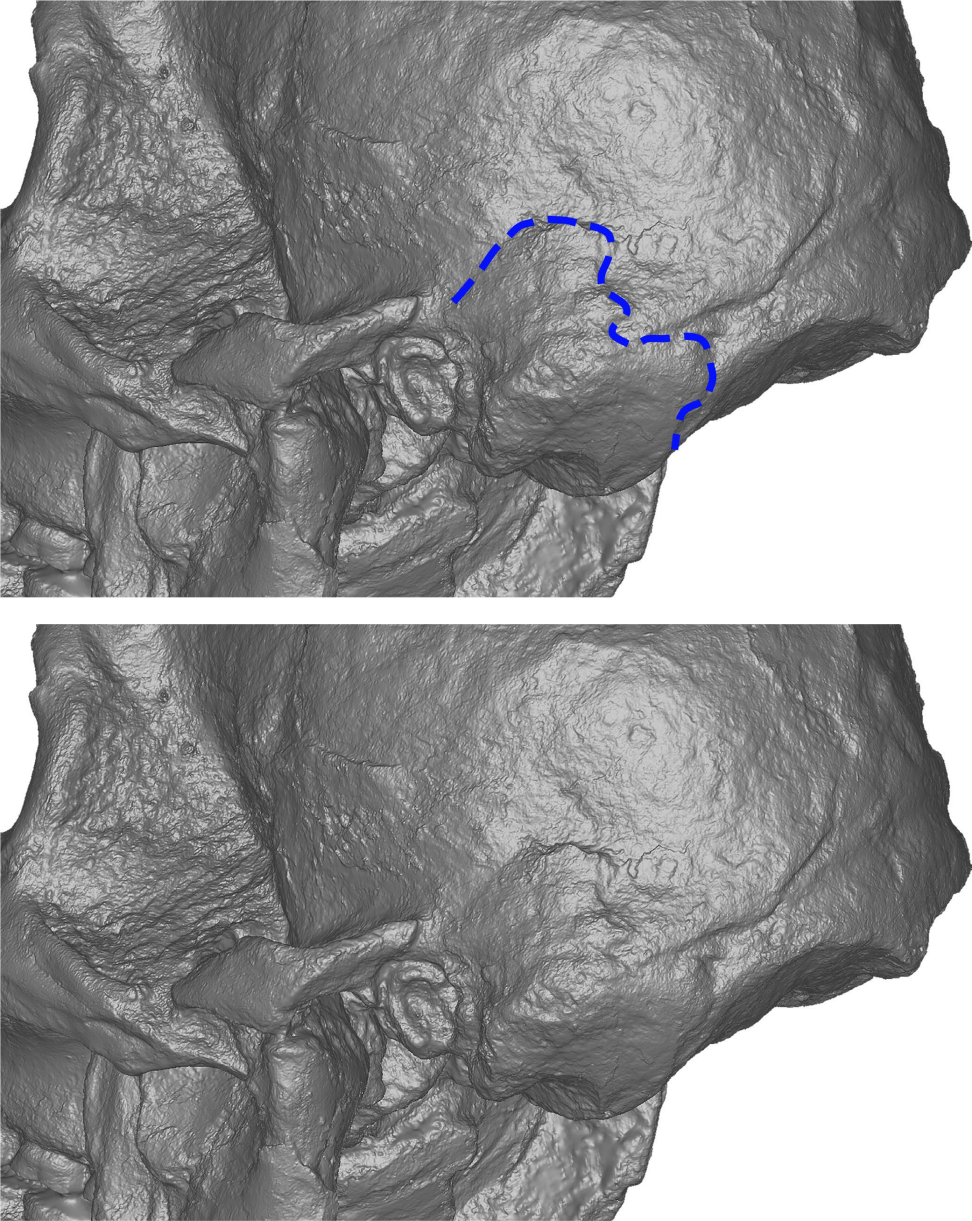
StW 573 in lateral view with second panel and blue line highlighting the presence of an asterionic notch.

Finally, StW 573 preserves an asterionic notch (Kimbel and Rak 1985) which is most clearly visible on the left side (Figure 2). Contrastingly, the morphology preserved by MLD 1 is not compatible with that specimen having had an asterionic notch, which is similar to the condition in Sts 5 and all other *A. africanus* specimens.

## 4 Discussion

The preserved morphology of StW 573 does not show close affinities with the type specimen of *Australopithecus prometheus*, MLD 1. Specifically, StW 573 differs from MLD 1 in having an asterionic notch, a sagittal crest at lambda, a large and well‐developed external occipital protuberance, a more inferiorly facing nuchal plane, and a long nuchal plane. In these respects, MLD 1 more closely resembles Sts 5 than StW 573. Furthermore, our analysis does not support Clarke and Kuman's (2019) assessment that MLD 1 and StW 573 share a lateral braincase wall that differs meaningfully from Sts 5. There is, therefore, no morphological justification for aligning StW 573 with MLD 1 to the exclusion of Sts 5, and on this basis the assignment of StW 573 to *A. prometheus* is not warranted. Indeed, there is no basis for suggesting that MLD 1 differs meaningfully from Sts 5, a consensus member of *A. africanus* . Thus, *A. prometheus* should, in our view, remain a junior synonym of *A. africanus* as conventionally recognized.

Clarke (1985, 1988, 2008, 2013), Clarke and Tobias (1995), Clarke and Kuman (2019) has consistently argued that there are two hominin species represented within the combined sample of gracile australopith fossils from Sterkfontein Member 4 and Makapansgat Limeworks, conventionally attributed to *A. africanus* . Our findings do not invalidate this hypothesis because the question of what a second species should be called is independent of whether a second species exists. It is not controversial to suppose that StW 573 may help to clarify whether that sample represents more than one species. If future studies demonstrate that fossils currently assigned to *A. africanus* can be sorted into two or more species, then *Australopithecus africanus* would retain naming priority for those specimens that remain assigned to the hypodigm including the Taung child. Specimens representing the second species will presumably be assigned to another existing or novel species. That species should not be *A. prometheus*.

If a novel species name is needed to accommodate StW 573, we suggest that this specimen be the holotype of a species named after the mythological figure who gave fire to humans, in one of South Africa's official languages. Such a name would provide etymological continuity with *A. prometheus*, named after the giver of fire from Greek mythology (Dart 1948). We refrain from formally erecting such a taxon here because it is more appropriate that a new species be named by the research team that has spent more than two decades excavating and analyzing the remarkable Little Foot specimen. We hope they will view our suggestion in this regard as well‐intentioned advice. In any case, there are no morphological traits that demonstrably align StW 573 with MLD 1 to the exclusion of other specimens conventionally assigned to *A. africanus*. Thus, StW 573 should not be attributed to *A. prometheus*.

## Author Contributions

**Jesse M. Martin:** conceptualization, investigation, writing – original draft, writing – review and editing, formal analysis, methodology. **Luca Morris‐Obst:** conceptualization, investigation, methodology, writing – review and editing, formal analysis. **A. B. Leece:** conceptualization, investigation, methodology, writing – review and editing, formal analysis. **Stephanie Baker:** writing – review and editing, project administration. **Andy I. R. Herries:** funding acquisition, writing – original draft, writing – review and editing, supervision. **David S. Strait:** conceptualization, funding acquisition, writing – original draft, methodology, writing – review and editing, formal analysis, supervision.

## Funding

This work was supported by the Australian Research Council (DP170100056).

## Conflicts of Interest

The authors declare no conflicts of interest.

## Data Availability

The data that support the findings of this study are available from University of the Witwatersrand. Restrictions apply to the availability of these data, which were used under license for this study. Data are available from the author(s) with the permission of University of the Witwatersrand.
